# Soluble PD-L1 in blood correlates positively with neutrophil and negatively with lymphocyte mRNA markers and implies adverse sepsis outcome

**DOI:** 10.1007/s12026-022-09302-y

**Published:** 2022-06-23

**Authors:** Marcus Derigs, Hendrik Heers, Susanne Lingelbach, Rainer Hofmann, Jörg Hänze

**Affiliations:** Department of Urology and Pediatric Urology, University Hospital, Philipps-University Marburg, Baldingerstr. 1, 35043 Marburg, Germany

**Keywords:** sPD-L1, Urosepsis, Polymorphonuclear neutrophils, PMNs, Lymphocytes, mRNA

## Abstract

Sepsis causes a myriad of immunological reactions that result in life-threatening alterations in the human body. Immunosuppression in sepsis is partly attributed to the programmed death receptor (PD-1) and its associated ligand (PD-L1) via the regulation of lymphocytes and neutrophils. Although the soluble forms of these proteins (i.e., sPD-1 and sPD-L1, respectively) are recognized as possible sepsis biomarkers, their functional implications are yet to be elucidated. Our research assessed the correlation between sPD-1 and sPD-L1 and blood mRNA markers and sepsis outcome. Blood samples of septic patients of urogenital origin versus control patients (both groups: *n* = 18) were analyzed. Blood serum sPD-1 and sPD-L1 levels were determined using the enzyme-linked immunosorbent assay (ELISA). The whole blood mRNA concentrations of PD-1, PD-L1, neutrophil markers (CEACAM8 and MPO), and T-lymphocyte markers (TCRβ, CD4 and CD8) were determined via reverse transcriptase quantitative PCR (RT-qPCR). sPD-L1 levels were significantly increased in septic patients when compared to the controls, whereas sPD-1 levels were unaltered. Patients with high sPD-L1 levels, as dichotomized to the median, had a significantly shorter survival rate than those with low sPD-L1 levels. The sensitivity/specificity characteristics of sPD-L1 proved significant for sepsis detection. Furthermore, sPD-L1 correlated with the mRNA concentrations of PD-L1, CEACAM, and MPO, as well as major inflammatory markers (C-reactive protein and procalcitonin). However, sPD-L1 negatively correlated with TCRβ, CD4, and CD8 mRNAs. sPD-L1 was found to be significantly increased in septic patients. Notably, sPD-L1 correlated with PD-L1 mRNA and neutrophil markers and was indicative of adverse outcomes.

**Supplementary Information:**

The online version contains supplementary material available at 10.1007/s12026-022-09302-y.

## Introduction

In sepsis, the immune response toward infection is disturbed with risks of collateral organ damage and multiorgan failure [1]. In this condition, the innate and adaptive immune responses are deregulated at multiple levels [2]. The most common sources of sepsis are of pulmonary (45%), abdominal (19–32%), or urogenital (9–31%) origin, with different incidences depending on the region [3]. Urosepsis results from urolithiasis (43%), benign prostatic hyperplasia (25%), urological cancer (18%), or other urological diseases (14%) [4]. This form of sepsis is diagnosed based on clinical symptoms, urine cultures, blood cultures, ultrasonography, and x-ray-based imaging.

A definitive viable biomarker for urosepsis is not yet available; however, procalcitonin (PCT) is currently the most widely employed urosepsis biomarker [5]. PCT appears to be a superior prognostic marker for general sepsis when compared to C-reactive protein (CRP), which has not yet been explicitly studied in urosepsis [6]. Moreover, interleukin-6 (IL-6) [7], wingless-type MMTV integration site family member 5a (Wnt5a) [8], and gelsolin [9] have been described as prognostic markers of urosepsis. Leukocytosis and leucopenia, which were previously part of the criteria for sepsis, were removed in the Third International Consensus Definitions for Sepsis and Septic Shock (Sepsis 3) due to their low prognostic validity for acute organ dysfunction in sepsis and mortality [1]. Interferon-gamma (IFN-γ) and IL-10 were ascribed prognostic and predictive value for sepsis outcome and therapy, as a low IFN-γ/IL-10 ratio was predictive for positive hydrocortisone therapy response in septic shock patients [10].

Recently, the programmed cell death receptor-1 (PD-1)—also called CD279—and one of its two primary ligands, programmed cell death ligand-1 (PD-L1)—also called B7-H1 or CD274—have gained importance with respect to the pathophysiology of sepsis and as a novel drug target for therapy [11]. PD-1 is expressed on active CD4^+^ and CD8^+^ T cells, B cells, natural killer cells, dendritic cells, and monocytes [12, 13]. Alternatively, PD-L1 is expressed on a wider array of immunocytes and also in tissues [14, 15].

Binding between PD-1- and PD-L1-expressing cells downregulates the T cell response to prevent collateral tissue damage after complete clearance of the initial infection. However, in sepsis, this process can be overactive and impair T cell function favoring immunosuppression [16]. In septic patients, an elevated expression of PD-1 and PD-L1 on T cells, monocytes, and natural killer cells, along with decreased T cell and natural killer cells activities, was demonstrated [17–19]. Recently, PD-L1 expression on neutrophils was shown to both favor the survival of neutrophils [20] and correlate with disease severity [18] in sepsis. Furthermore, increased PD-L1 expression on monocytes was shown to be an independent predictor of 28-day mortality in septic shock patients [19]. In murine sepsis models, PD-1 deficiency displayed reduced sepsis lethality by balancing pathogen clearance and inflammatory cytokine production [21]. Subsequently, anti-PD-1 and anti-PD-L1 antibodies were utilized, resulting in the improvement of survival in murine sepsis models through lymphocyte depletion reduction and apoptosis inhibition [21, 22]. In sepsis patients, the decreased phagocytic function of neutrophils and monocytes was restored ex vivo using anti-PD-1 and anti-PD-L1 antibodies [18]. Concurrently, the anti-PD-1 antibody, Nivolumab, demonstrated a favorably safety profile and alleviated immunosuppression when administered to sepsis patients in a phase 1 study [23].

In addition to the membrane-bound forms of PD-1 and PD-L1, their soluble forms (i.e., sPD-1 and sPD-L1, respectively) have been identified in blood. These proteins are derived from certain truncated splice variants [24–27]. Notably, sPD-L1 can also be shed from membrane-bound PD-L1 by proteolysis [28]. Consequently, sPD-1 and sPD-L1 levels are related to their cellular expression levels. Functionally, sPD-1 and sPD-L1 can act antagonistically in intercellular PD-1/PD-L1 signaling [25]. Previous studies have demonstrated diverging sPD-1 and sPD-L1 levels in sepsis patients. Although sPD-1 and/or sPD-L1 levels were found to be increased in sepsis patients in some studies [29, 30], other studies reported decreased sPD-1 levels [31] or no differences in sPD-1 and sPD-L1 in sepsis patients versus healthy controls [32]. In these studies, the heterogeneous septic sources were not differentiated, and urosepsis was a minor focus [29, 32].

Against this background, we aimed to expound the meaning of sPD-1 and sPD-L1 in urosepsis patients, focusing on the expression sources, regulation, and characteristics as biomarkers. Therefore, we determined the serum sPD-1 and sPD-L1 levels and mRNAs of PD-1 and PD-L1 in whole blood of urosepsis patients and compared them to non-septic controls. We further considered certain blood mRNA markers as surrogates for the matched profiling of neutrophils and lymphocytes. Lastly, we analyzed patients’ survival to evaluate sPD-1 and sPD-L1 as possible prognostic biomarkers in relation to CRP and PCT.

## Methods

### Patients and controls

This prospective observational single-center cohort study was conducted with the approval of the Ethics Board of the University Hospital of Marburg, Germany. Informed consent was obtained from all study subjects or their legal representatives. Recruitment took place between November 2018 and August 2020. The study subjects consisted of patients with urosepsis (*n* = 18) treated in the urological intermediate care (IMC) ward or one of the hospital intensive care units. The inclusion criteria for the patients with urosepsis were based on the “Sepsis-3” definition of the “Third International Consensus Definitions for Sepsis and Septic Shock” and comprised only septic patients with urogenital foci of infections [1]. Hence, next to positive urine and/or blood cultures, patients with urosepsis needed to score at least two points on the Sequential Organ Function Assessment (SOFA) Score. Study subjects were compared to control subjects (*n* = 18) that were treated in the urological ward for benign urological disease. Control subjects were otherwise healthy and had negative urine cultures. The exclusion criteria for urosepsis and control subjects were as follows: (1) Pre-existing active neoplastic disease; (2) active autoimmune disease; (3) active infectious disease; (4) HIV infection; (5) pregnancy; (6) active hormonal or immunosuppressive treatment; (7) liver disease; (8) end-stage renal disease; (9) post-operative urosepsis; and (10) lack of informed consent.

### Data collection

The baseline demographic and clinical data which included age, gender, vital signs (heart rate, blood pressure, respiratory rate, and temperature), altered consciousness state, comorbidities, medical history, routine blood tests (including PCT, CRP, liver, kidney, and coagulation function), and bacteriologic tests of the patients’ urine and blood were recorded upon admission. Laboratory data were collected for up to 18 days. The baseline disease severity was assessed using the Acute Physiology and Chronic Health Evaluation (APACHE) II, the SOFA, and the quick SOFA (qSOFA) score. Blood samples of the control subjects were taken during routine blood drawing. We ensured follow-up by obtaining patient contact information and contacting patients by telephone. Where feasible, the patients’ clinical course, outcomes, and secondary infections were documented after 1 year.

### Analyses of blood samples

CRP, creatinine, leukocyte count, and PCT were determined in the central laboratory of the hospital. The values for CRP, creatinine, and leukocyte count were available for all study subjects (*n* = 18) and control subjects (*n* = 18). The PCT values were not obligatorily determined for hospitalized patients and were available for (*n* = 14) of the study subjects at admission. In the research laboratory of the Urology Department, sPD-L1 and sPD-1 and RNA analytics of whole blood were performed. sPD-L1 was measured for all patients with urosepsis and (*n* = 18) control patients (*n* = 18) by commercial ELISA kits (sPD-L1: R&D-Systems, Quantikine human B7-H1/PD-L1 DB7H10; sPD-1: R&D-Systems, DY1086/Duo ancillary reagent kit 2 DY008) according to the manufacturers’ instructions. The detection limits of sPD-L1 or sPD-1 were determined by the actual ELISA standard curves based on the applied optical hardware and analytical software (Molecular Devices Emax, Soft Max Pro6.4).

RNA from whole blood was collected and extracted with the PAXgene system (PreAnalytiX, Qiagen: 763,134). Whole blood RNA analytics requires specific sampling protocols that are not routinely covered for hospitalized patients at admission. Therefore restrictively, whole blood RNA samples were available from patients with urosepsis (*n* = 10) and control patients (*n* = 11).

### Reverse transcriptase quantitative PCR (RT-qPCR)

RNA (0.5 µg) treated with DNAse followed by heat denaturation (70 °C, 5 min) was submitted to cDNA synthesis with random hexamers and M-MLV reverse transcriptase. The cDNA was further analyzed by RT-qPCR (IQ5, Biorad, Germany) with SYBR green (ThermoScientific, UK) detection. Cycling conditions were 95 °C, 7.5 min, followed by 40 cycles (95 °C for 15 s; 58 °C for 30 s; 72 °C for 30 s). Melting curve analysis was performed by temperature increments of 0.5 °C every 10 s from 60 to 95 °C. Target mRNA levels are displayed as -∆Ct values (log 2-scale) normalized to TATA-binding protein (TBP) mRNA as reference [33]. The primer sets (Biomers GmbH, Germany) were derived from GenBank sequence entries and selected by Primer-Blast (NCBI National Center for Biotechnology Information). The corresponding sequences are listed (Supplementary **Table **S1).

### Statistical analysis

The statistical analysis was carried out with Prism 8 (GraphPad Software, La Jolla, CA, USA). Spearman’s test was used to analyze the correlation between sPD-L1 and inflammatory markers as well as mRNA of important molecules. The comparison between the two groups was analyzed using the Mann–Whitney *U* Test. Survival was analyzed via Kaplan–Meier analysis. Biomarker characteristics were calculated by receiver operating characteristic (ROC) curve analysis and determination of the area under the curve (AUC).

## Results

### Patient demography

**Fig. 1:**
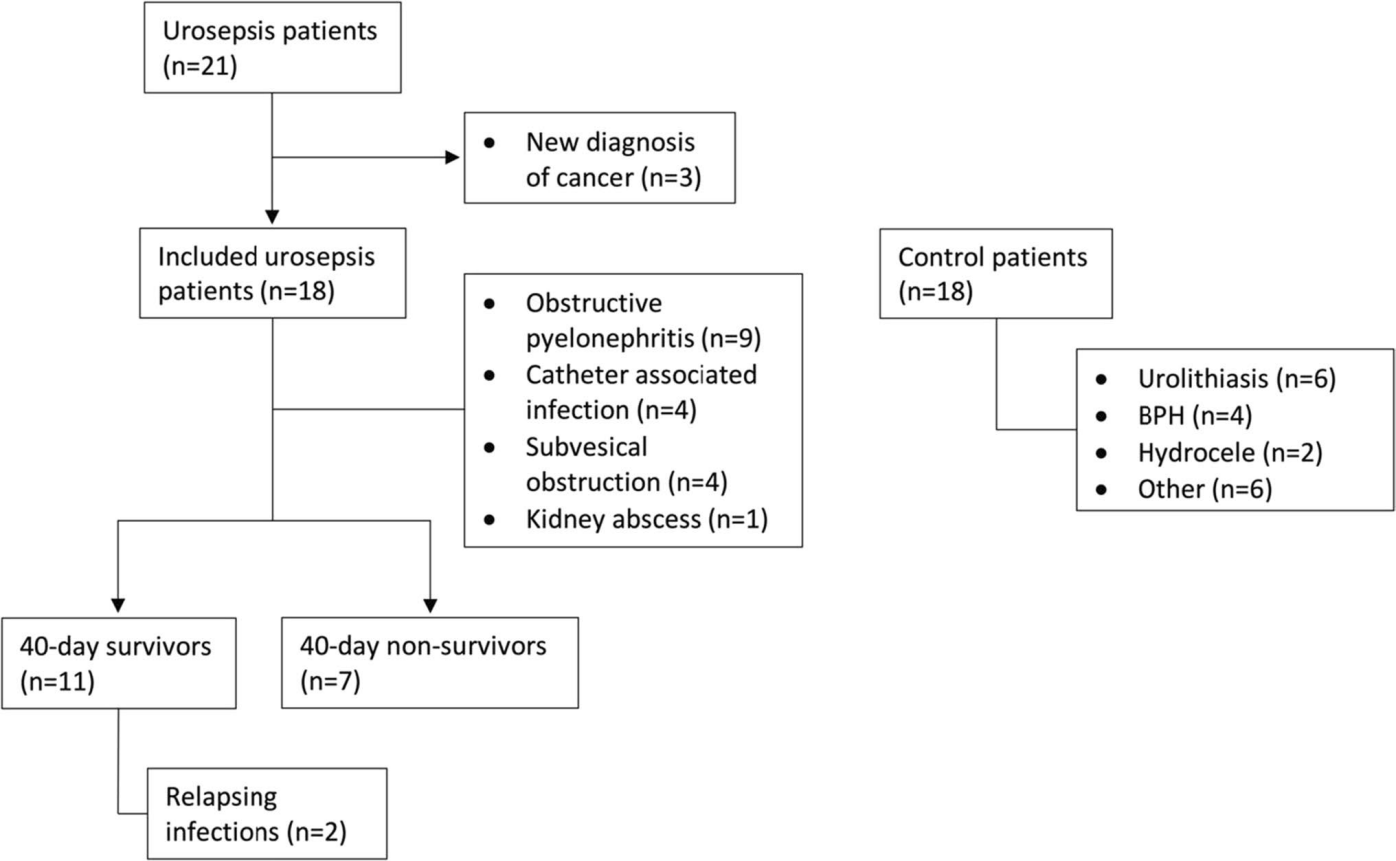
Patient selection flow chart

We screened 21 patients with urosepsis, of which three were excluded due to newly diagnosed bladder cancer. Hence, we enrolled 18 patients with urosepsis and 18 control patients **(Fig. **1**)**. Three patients with urosepsis died during their hospital stay and seven patients with urosepsis died within 40 days of admission. Eleven patients with urosepsis were still alive at 1-year follow-up. Two of the survivors suffered from relapsing infections. The control patients were treated for benign urological diseases, most commonly urolithiasis and benign prostatic hyperplasia (BPH**; Fig. **1). The demographic data, severity scores, blood values, pre-existing conditions, and microbiological tests of the urosepsis and control patients are presented, and significant differences between urosepsis and control patients are indicated (**Table **1).

**Table 1:** Characteristics of urosepsis and control patients

Baseline characteristics	Urosepsis patients			Control patients	p-value
	Survivor	Non-Survivor	Total		
Number	11	7	18	18	/
Age, years, mean ± SD	73 ± 14.8	85 ± 5.7	78 ± 13.3	63 ± 16.3	0.0034
Male, n (%)	4 (36)	5 (71)	9 (50)	15 (83)	0.034
In-hospital lethality, n (%)	/	3 (42)	3 (17)	/	/
Relapsing infections, n (%)	2 (18)	/	2 (11)	/	/
Scoring systems:					
APACHE-II, mean ± SD	21.4 ± 6.9	21.6 ± 2.4	21.4 ± 5.5	/	/
SOFA, mean ± SD	4 ± 3.3	5.3 ± 2.1	4.5 ± 2.9	/	/
qSOFA, mean ± SD	2 ± 0.6	2 ± 0.6	2 ± 0.6	/	/
Lab values on day of admission:					
Leucocytes G/l, mean ± SD	17.8 ± 6.3	19 ± 11.6	18.3 ± 8.4	8 ± 3.9	0.0001
CRP mg/dl, mean ± SD	169 ± 100	218 ± 111	188 ± 104	11 ± 17.8	0.0001
PCT ug/l, mean ± SD	19 ± 20	61.7 ± 44.8	40.5 ± 40	/	/
Creatinine mg/dl, mean ± SD	1.8 ± 1	4.5 ± 3.8	2.9 ± 2.8	1 ± 0.3	0.0002
Pre-existing conditions:					
Hypertension, n (%)	5 (45)	2 (29)	7 (39)	5 (28)	0.47
Diabetes, n (%)	2 (18)	2 (29)	4 (25)	5 (28)	0.7
Parkinson, n (%)	2 (18)	1 (14)	3 (17)	/	/
Positive culture:					
Gram positive, n (%)	2 (18)	2 (29)	4 (25)	/	/
Gram negative, n (%)	11 (100)	5 (71)	16 (89)	/	/

Abbreviations: number (n), standard deviation (SD); Acute Physiology and Chronic Health Evaluation (APACHE); Sequential Organ Failure Assessment (SOFA); quick SOFA score (qSOFA); C-reactive protein (CRP), procalcitonin (PCT). The p-value was calculated from total urosepsis versus control patients (Mann–Whitney U Test or Chi-square test)

### sPD-L1 and sPD-1 concentrations in sepsis and control patients

**Fig. 2:**
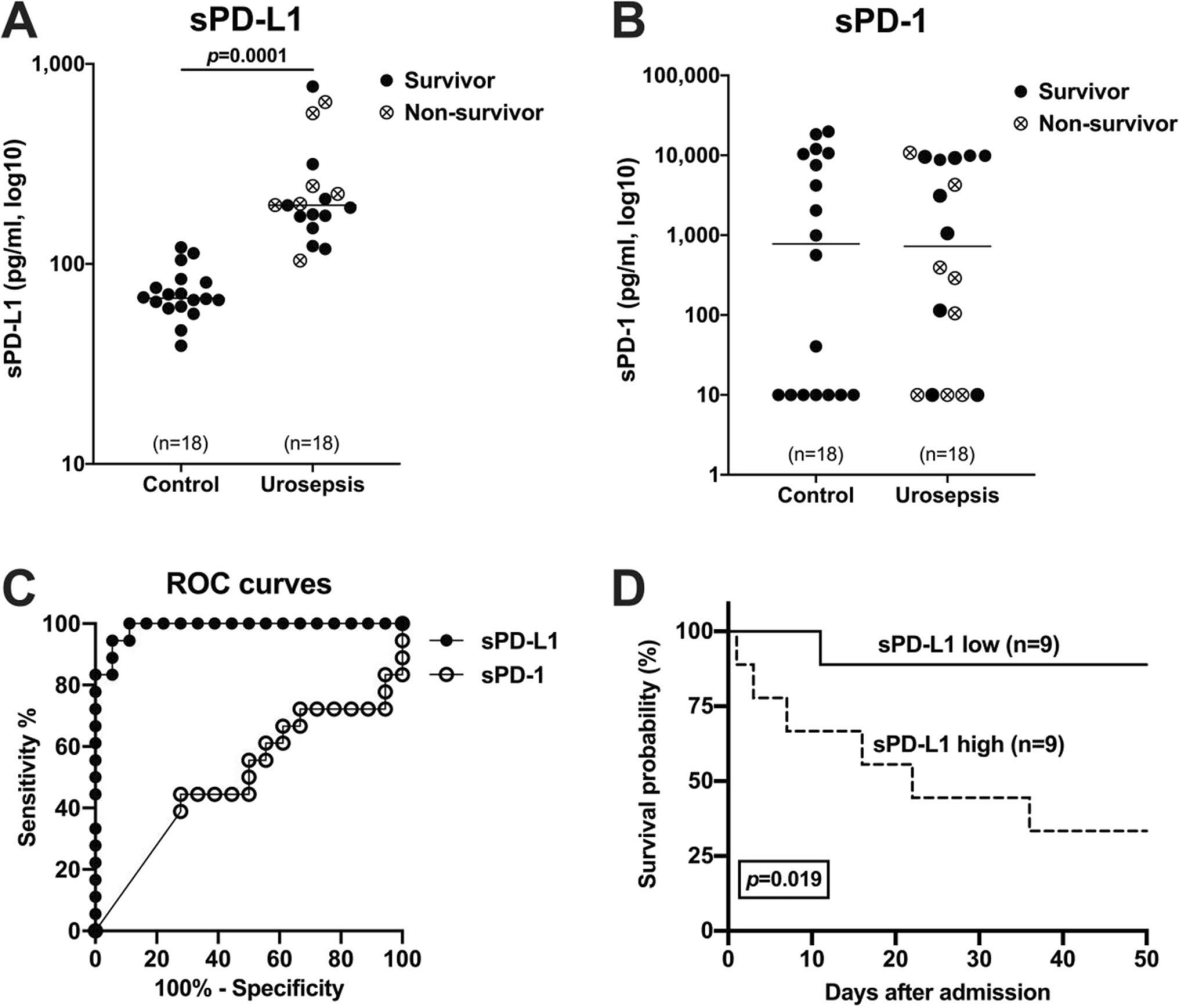
Analysis of sPD-L1 (**A**) and sPD-1 (**B**) in urosepsis (black) and control (red) patients. The median of each group is displayed by a horizontal line with numbers (n) and *p* value (Mann–Whitney U Test). Survivors are labeled by filled circles and non-survivors by crossed circles. (**C**) Analysis of control and urosepsis patients by receiver operating characteristic (ROC) curves for sPD-L1 (AUC 0.988, *p* < 0.0001) and sPD-1 (0.502, *p* = 0.987). (**D**) Kaplan–Meier survival curves of urosepsis patients with high (*n* = 9) and low sPD-L1 level (*n* = 9) divided according to the median. Urosepsis patients with high sPD-L1 level had significant (*p* = 0.019) shorter survival than those with low sPD-L1 level

The serum levels of sPD-L1 were significantly higher in patients with urosepsis than in control patients (**Fig. **2A; *p* < 0.0001, *n* = 18), whereas sPD-1 did not differ significantly (**Fig. **2B; *p* = 0.9932, *n* = 18). In addition, we tested sPD-1 and sPD-L1 with respect to sensitivity/specificity for discriminating between sepsis patients and control patients (ROC curve) in these cohorts. sPD-L1 exhibited significant value as a biomarker for sepsis (**Fig. **2C; AUC = 0.987, *p* < 0.0001), whereas sPD-1 did not (**Fig.** 2C; AUC = 0.502, *p* = 0.987). In contrast, the AUC values were also significant for CRP (**Fig. **S1; AUC = 0.987, *p* < 0.0001) and leucocytes (**Fig. **S1; AUC = 0.895, *p* < 0.0001). The subset of patients with urosepsis was further analyzed with respect to survival. Dichotomizing by the median, the high sPD-L1 level group exhibited significantly shorter survival than the low sPD-L1 group (**Fig. **2D; *p* = 0.019, *n* = 18). In contrast, for sPD-1 no significant difference in survival between the high and low sPD-1 groups was apparent (*p* = 0.101). In addition, no significant differences in survival were observed for the respective high versus low leucocyte, CRP, and PCT groups (**Fig. **S2).

### sPD-L1 and sPD-1 in relation to mRNA markers in whole blood

**Fig. 3:**
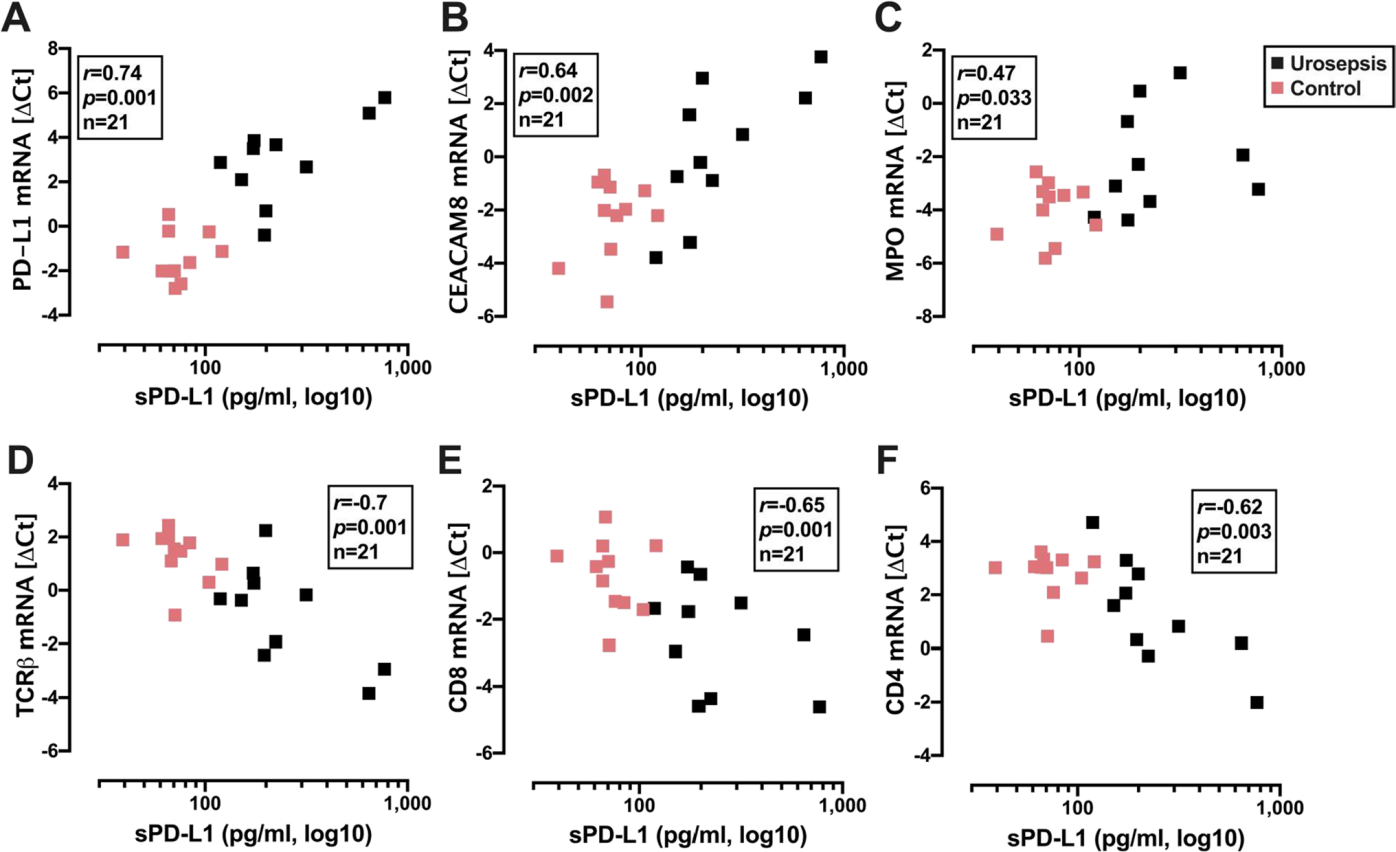
Correlation analyses of sPD-L1 with PD-L1 mRNA and several blood cell mRNA markers. PD-L1 mRNA (**A**); neutrophil markers: CEACAM8 mRNA (**B**) and MPO mRNA (**C**); T-lymphocyte markers: TCRβ mRNA (**D**), CD8 mRNA (**E**), and CD4 mRNA (**F**). Urosepsis patients are labeled black and control patients red. The Spearman correlation coefficient, *p* value, and *n* number are shown

Next, we analyzed possible associations of several mRNA markers in whole blood to sPD-L1 and sPD-1. We observed a significant correlation between sPD-L1 and PD-L1 mRNA (**Fig. **3A; *p* = 0.001, *r* = 0.74), but not between sPD-1 and PD-1 mRNA (*p* = 0.73, *r* =  − 0.08).

In the literature, the neutrophil/lymphocyte ratio was described as a robust biomarker for sepsis [34]. Therefore, we also determined mRNA markers of neutrophils (CEACAM8 and MPO; **Fig. **3B-C) and lymphocytes (TCRβ, CD8, and CD4; **Fig. **3D-F). Interestingly, we found a tight positive correlation between sPD-L1 and CEACAM8 mRNA (*r* = 0.64, *p* = 0.002) and MPO (*r* = 0.47, *p* = 0.033). Contrastingly, we observed a significant negative correlation between sPD-L1 and TCRβ mRNA (*r* =  − 0.7, *p* = 0.001), CD8 (*r* =  − 0.65, *p* = 0.001), and CD4 (− 0.62, *p* = 0.003).

**Fig. 4:**
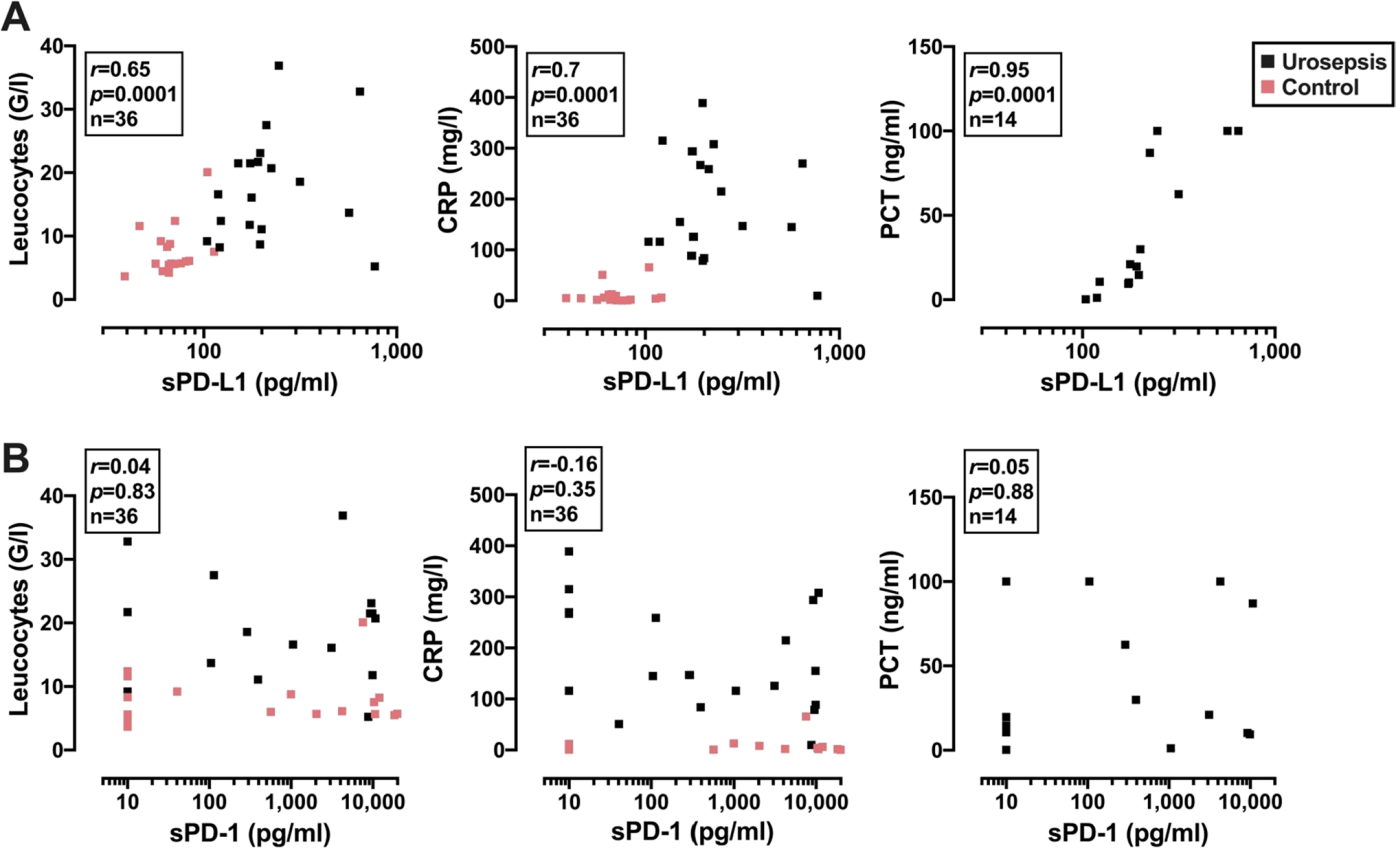
Correlation analyses of sPD-L1 (**A**) and sPD-1 (**B**) with leucocyte number and the inflammatory markers C-reactive protein (CRP) and procalcitonin (PCT). Urosepsis patients are labeled black and control patients red. The Spearman correlation coefficient, *p* value, and *n* number are shown

Finally, we compared the levels of sPD-L1 and sPD-1 to established inflammatory markers. We observed a significant positive correlation between sPD-L1 and leucocyte count, CRP, and PCT; in contrast, no correlation was found between these markers and sPD-1 (**Fig. **4).

## Discussion

Soluble immune checkpoint components derived from PD-1 and PD-L1 during sepsis are not adequately understood. sPD-1 and sPD-L1 may monitor changes in the blood caused by systemic infection, which can be exploited as diagnostic or prognostic markers in sepsis. We investigated the blood of patients for matched case–control analyses of serum sPD-1 and sPD-L1, as well as related mRNA targets in whole blood. The results of our study are as follows: (i) sPD-L1 was increased in the cohort of sepsis patients versus control patients; (ii) sPD-L1 correlated with PD-L1-mRNA in whole blood; (iii) sPD-L1 displayed significant potential as a sepsis biomarker due to its association with adverse outcomes; (iv) sPD-L1 correlated positively with mRNA markers of neutrophil granulocytes and negatively with mRNA markers of T-lymphocytes; and (v) contrary to sPD-L1, sPD-1 levels varied arbitrarily in both urosepsis and control patients.

### Blood sPD-1 and sPD-L1 values in urosepsis versus sepsis

Different pathogenic infection sources can trigger systemic sepsis with similar immunologic and clinical manifestations. The source of infection is related to different pathogens that can target different pattern recognition receptors (PPRs) of the innate immune system, with characteristic tissue and cell-specific expression levels [35]. The respective profile of induced cytokines can be linked to partially discrete adaptive immune responses including co-inhibitory and co-stimulatory regulators affecting sepsis outcomes. For biomarker analysis, it is particularly relevant to consider and differentiate the infection site. Only a handful of studies in this field have explored urosepsis as a separate entity from general sepsis. To date, PCT is the best established distinct marker for urosepsis [9]. We directed our attention to the immunomodulatory molecules, PD-1 and PD-L1, as they have emerged as important contributors to T cell exhaustion in sepsis. It was shown that an increased expression of PD-1 on T cells [8], PD-L1 on monocytes [19, 36], or PD-L1 on neutrophils [37] was related to a worse prognosis in septic shock patients. Our data suggest the importance of sPD-L1 but not sPD-1 in patients with urosepsis and correlate with a study, which included only 3% of urosepsis patients that observed elevated sPD-L1 in patients with general sepsis [29]. The significance of sPD-1 as a biomarker in urosepsis and sepsis is less clear. In the literature, one study revealed decreased sPD-1 levels [31], whereas another study displayed increased levels [30].

### sPD-L1 and PD-L1 mRNA expression in peripheral blood cells

The correlation between sPD-L1 and PD-L1 mRNA concentrations in whole blood RNA suggests that sPD-L1 is post-transcriptionally processed and released from blood cells. Since sPD-L1 positively correlates with neutrophilic markers (CEACAM8 and MPO) and neutrophils constitute the majority (60–70%) of leucocytes, neutrophils may constitute the major source of sPD-L1 in blood. Based on these observations, we suggest that sPD-L1 is related to the neutrophil count and may be regulated by pro-inflammatory crosstalk with lymphocytes. Accordingly, RNA-seq data from normal blood cells combined with cell sorting exhibited the highest levels of PD-L1 mRNA in neutrophils, followed by T cells, and monocytes [25]. The PD-L1 mRNA levels were determined by the number of reads of different PD-L1 mRNA variants, demonstrating that neutrophils and CD8^+^ T cells had substantially higher fractions of PD-L1 mRNA-positive cells than monocytes and other investigated peripheral blood cells [25]. Blood sPD-L1 may indicate the action of several altered cytokines in sepsis, such as IFN-γ and IL-10 [10]. Notably, besides the well-established IFN-γ-dependent induction, IL-10 was additionally shown to induce PD-L1 [38, 39]. Both cytokines were induced by in vitro challenge of whole blood with bacterial burden [10]. Interestingly, in an experimental abdominal murine sepsis model, neutrophil IL-10 production was induced by IFN-γ from CD4^+^ T cells, thereby dampening local inflammation [40].

### Possible functions of sPD-L1 in relation to neutrophils and lymphocytes

An increase in neutrophils and a decrease of lymphocytes are known inflammatory immune cell responses toward infection [41]. The positive correlation between sPD-L1 and neutrophil and its negative correlation with lymphocyte markers may reflect this phenomenon. However, the involvement of sPD-L1 in these shifts of neutrophilic and lymphocytic shifts is speculative. Possibly, agonistic sPD-L1 released from neutrophils may trigger apoptosis in PD-1^+^ lymphocytes. A related process has been suggested in immuno-oncology, in which sPD-L1 derived from cancer cells demonstrated interference with cellular PD-L1/PD-1 signaling. Particularly, sPD-L1 may deliver an apoptotic signal to CD8^+^ T cells, thereby weakening the anti-tumor immune cell response [28]. With regard to sepsis, another neutrophilic PD-L1 function was previously demonstrated [20, 42]. Cellular PD-L1 exerts a pro-survival function in neutrophils that appears independent of its interaction with PD-1. Specifically, neutrophils that were exposed to inflammatory signals, including IFN-γ and LPS, induced PD-L1 expression. By mediating the PI3K/AKT phosphorylation pathway, PD-L1 inhibited neutrophil apoptosis, thereby favoring their survival. Accordingly, PD-L1 silencing prolonged survival in a sepsis model involving mice by ameliorating lung dysfunction. In this regard, it would be interesting to explore whether the induction of PD-L1 expression by IFN-γ and LPS in neutrophils also affects sPD-L1 release.

### sPD-L1 as a predictive biomarker for sepsis and in relation to inflammatory markers

The study results reveal that urosepsis patients with high sPD-L1 levels had a shorter survival time (**Fig. **2D). These results are consistent with published data correlating increased sPD-L1 levels with lower survival rates in general sepsis [29]. Additionally, two other studies separately demonstrated that increased PD-L1 expression on monocytes was a predictor of disease mortality in general sepsis [19, 43]. Together with our finding of a positive correlation between PD-L1 mRNA and sPD-L1 levels (**Fig. **3A), this strengthens the hypothesis that the membrane-bound PD-L1 is one source of sPD-L1 [27]. Nevertheless, no correlation was found between sPD-L1 levels with the major severity scores of sepsis (qSOFA, SOFA and APACHE-II) in these patients (data not shown). We deduce that blood sPD-L1 levels do not necessarily reflect the matched severity scores due to varying sequences during sepsis. In partial accordance with our study, Liu et al. observed increased levels of sPD-L1 and sPD-1 in sepsis patients compared to the control group [29]. Similar to our study, patients with high sPD-L1 levels had a shorter survival time. Another study also displayed increased sPD-1 levels in septic patients [30]. In marked contrast to these studies, our research did not reveal elevated sPD-1 levels in urosepsis patients with or without septic shock. Our study’s control group more frequently had higher sPD-1 levels than the sepsis group, but without significance (**Fig. **2B) compared to a previous study stating that healthy individuals do not express sPD-1 [44]. Assuming that sPD-1 levels correlate with PD-1 expression, our finding of unaltered sPD-1 levels in patients with urosepsis confirms the result of one study, which showed that PD-1 immune cell expression in septic patients did not correlate with any clinical parameters [18]. However, other studies demonstrated increased expression of PD-1 on CD4^+^ and CD8^+^ T cells in septic patients [17, 45]. Speculatively, the increased expression of membrane-bound PD-1 may not be solely linked to sPD-1.

Different distributions in sepsis etiology as reflected, to a major part, by respiratory and abdominal infections compared to urinary tract infections in our study become apparent [29, 32]. Another study by Zhao et al. found increased levels of sPD-1 and sPD-L1 in sepsis versus control patients where the sPD-1 levels had a predictive value for the severity sepsis [46]. This study also fractioned patients with respiratory and abdominal infections into a single group. Yu et al. found that in patients with pancreatitis, sPD-1 was particularly increased in the blood of patients with infectious complications [47]. Bakshiani et al. observed an sPD-1 correlation with the inflammation markers CRP and PCT [48].

## Limitations

The major limitation of our study is the lack of relation between sPD-1 and sPD-L1 and neutrophil and lymphocyte blood cell counts. Nevertheless, the positive correlation analyses of sPD-L1 to PD-L1 mRNA and neutrophilic markers (CEACAM8 mRNA and MPO mRNA) and its negative correlation to lymphocyte markers (TCRβ mRNA, CD4 mRNA, and CD8 mRNA) in whole blood RNA represent a new finding. Our study suggests that these selected blood-subset cell-mRNA markers are exploitable as surrogates for their respective peripheral blood cell counts. PD-L1 expression and sPD-L1 release should be directly analyzed in the isolated blood neutrophils and lymphocytes of septic and non-septic patients.

## Conclusions

sPD-L1 was significantly increased in sepsis patients. Moreover, sPD-L1 correlated positively with PD-L1 mRNA and neutrophil mRNA markers in whole blood and indicated an adverse sepsis outcomes.

## Supplementary Information

Below is the link to the electronic supplementary material.

Supplementary file1 (DOCX 306 kb)
