# Supplementary material for: Soluble PD-L1 in blood correlates positively with neutrophil and negatively with lymphocyte mRNA markers and implies adverse sepsis outcome
Source: Immunol Res. 2022 Jun 23;70(5):698–707. doi: 10.1007/s12026-022-09302-y (PMC9499885; doi:10.1007/s12026-022-09302-y)
Supplement: Supplementary file 1 — Supplementary file1 (DOCX 306 kb) [file 12026_2022_9302_MOESM1_ESM.docx]

# SUPPLEMENTAL DATA

| **Target**  Gene ID/Synonym | **GenBank/NCBI** Accession | **Primer sequence (5' - 3')**  Forward (F)/Reverse (R**)** |
| --- | --- | --- |
| CD274/PD-L1 | NM_014143 | F: TGTGACCAGCACACTGAGAATCA |
|  |  | R: TGGAGGATGTGCCAGAGGTAGT |
| PDCD1/CD279/ PD-1 | NM_005018 | F: CCGGCCAGTTCCAAACCCTG |
|  |  | R: TGCGCCTGGCTCCTATTGTCC |
| CEACAM8 | NM_001816 | F: CCCCAGGACCCTCGTGGCTA |
|  |  | R: GACGTTCCGCATCAGCAGGGA |
| MPO | M19507 | F: TGGCGTCAACTGCGAGACCA |
|  |  | R: GCGCGTTGATCTGGTTGCGG |
| TRB/TCRβ | NG_001333 | F: TATGTTTTGGTATCGTCAGTTCCCG |
|  |  | R: CACTGCAGATGTAGAAGCTGCTGTC |
| CD8A | NM_001768 | F: AGCCACTTCGTGCCGGTCTT |
|  |  | R: CCAAGGGCGCCCAGATGTAGA |
| CD4 | NM_000616 | F: TGCTAGTGTTCGGATTGACTGCCA |
|  |  | R: ACAGTGCATGTCCAGGTGCCA |
| TBP | NM_003194 | F: TCATGAGGATAAGAGAGCCACGAAC |
|  |  | R: TAGGAAACTTCACATCACAGCTCCC |

# Table S1: List of the primer sequences for qRT-PCR

#
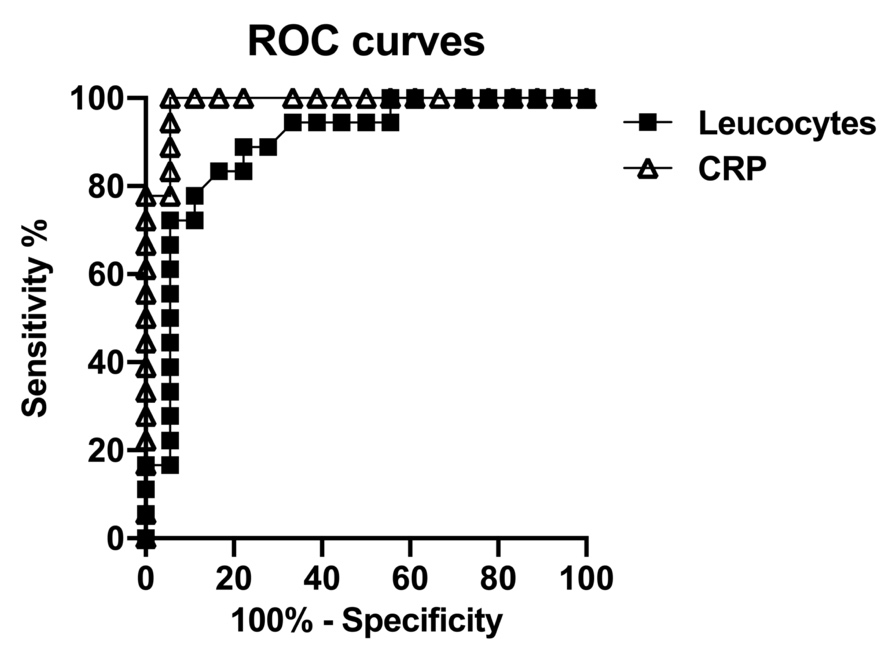


# Figure S1: Analysis of control and urosepsis patients by receiver operating characteristic (ROC) curves for leucocytes (AUC 0.895, p<0.0001) and CRP (0.987, p=0.0001).

#
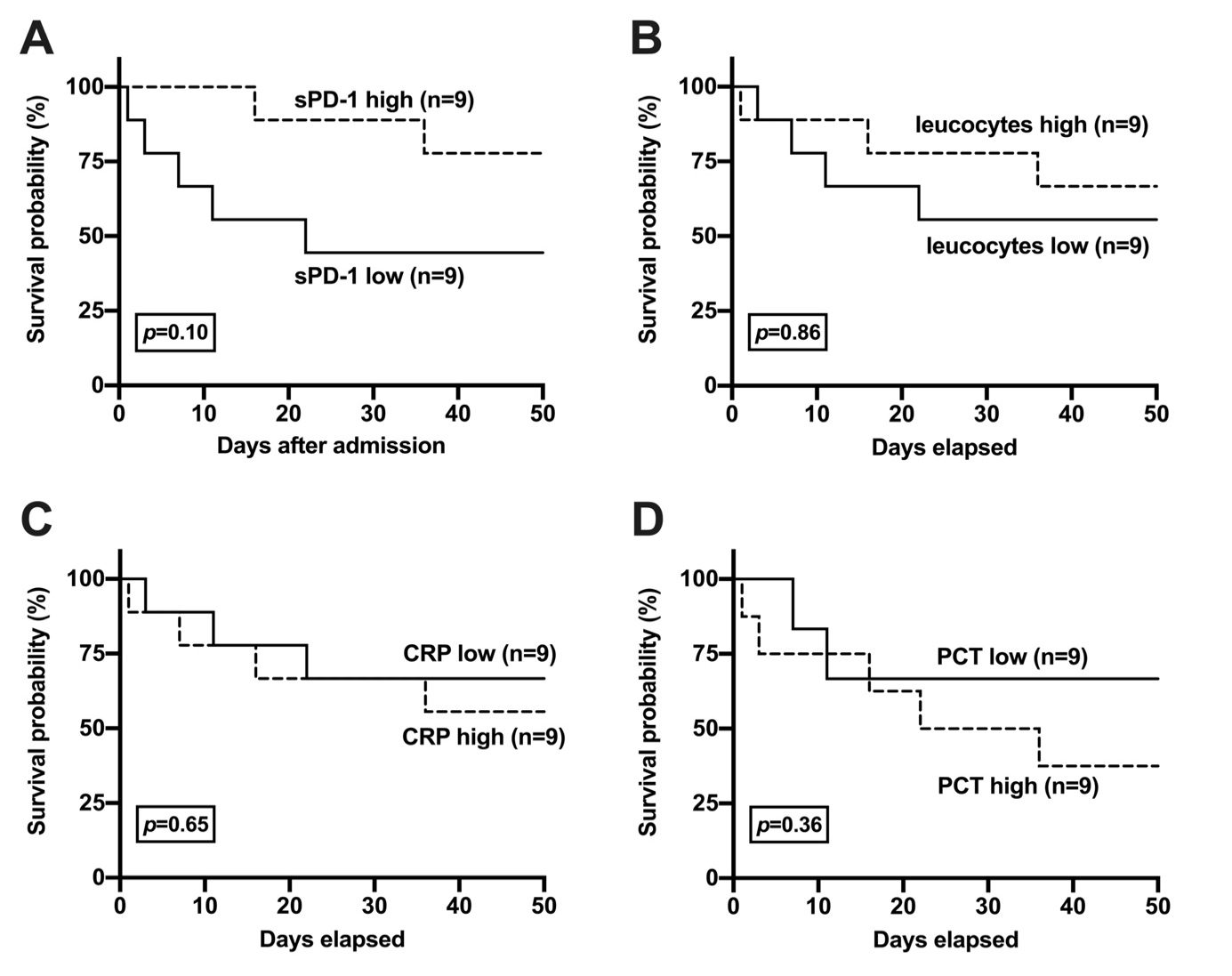


# Figure S2: Kaplan-Meier survival curves of urosepsis patients in regard to high and low levels of sPD-1 (A), leucocytes (B), CRP (C) and PCT (D), divided according to the median. In contrast to sPD-L1, none of these markers showed a significant correlation to survival probability.
